# Essential Oil Compounds of Andaliman (*Zanthoxylum acanthopodium* DC.) Fruit Varieties and Their Utilization as Skin Anti-Aging Using Molecular Docking

**DOI:** 10.3390/life13030754

**Published:** 2023-03-10

**Authors:** Endang Kintamani, Irmanida Batubara, Cecep Kusmana, Tatang Tiryana, Edi Mirmanto, Shadila F. Asoka

**Affiliations:** 1 Department of Silviculture, Faculty of Forestry and Environment, IPB University, Bogor 16680, Indonesia; 2 Research Center for Ecology and Ethnobiology, National Research and Innovation Agency, Cibinong 16911, Indonesia; 3 Department of Chemistry, Faculty of Mathematics and Natural Sciences, IPB Univeristy, Bogor 16680, Indonesia; 4 Tropical Biopharmaca Research Center, Institute of Research and Community Servies, IPB University, Bogor 16128, Indonesia; 5 Department of Forest Management, Faculty of Forestry and Environment, IPB University, Bogor 16680, Indonesia

**Keywords:** D-limonene, GC-MS, geranyl acetate, rejuvenation, Sihalus variety

## Abstract

Exposure to UV/infrared (IR) radiation is the main extrinsic factor that changes skin morphology and affects the increase in reactive oxygen species (ROS) in skin aging. Ten varieties of andaliman (*Zanthoxylum acanthopodium* DC.) fruit are presumed to have skin anti-aging compounds via an enzyme-inhibition mechanism. This study aims to compare ten essential oils (EOs) of andaliman fruit varieties, group them according to their varieties, and obtain the chemical components that can be used as potential skin anti-aging agents using molecular docking. EOs were isolated by hydrodistillation, and the determination of the chemical compounds was performed using gas chromatography-mass spectrometry (GC-MS). Using the Orange data mining software, a heatmap was used for grouping and showing the abundance of the compounds of ten varieties. Finally, molecular docking was conducted using the software AutoDockTools 1.5.7. There were 97 chemical components in the ten EOs of andaliman fruit varieties, with the main chemical components being geranyl acetate (29.87%) and D-limonene (26.49%), and they were grouped into three clusters. The chemical components that are prospective candidates as skin anti-aging agents are geranyl acetate and D-limonene, found in abundance in the Sihalus variety of andaliman fruit. These can be developed for applications in the pharmaceutical industry.

## 1. Introduction

Skin aging is a multivariable process that influences every step of biological life [1]. Exposure to UV/infrared (IR) radiation is one of the main extrinsic factors that change skin morphology, influencing the increase in reactive oxygen species (ROS) bound in the skin in dissimilar indications. Aged skin is marked by epidermal depletion, wrinkling, and a decrease in elasticity [1]. UV radiation can also increase the risk of skin cancer. Aging can also be triggered by the activity of enzymes in the body. The enzymes involved in skin aging include collagenase, hyaluronidase, elastase, and tyrosinase. Collagenase can cause skin aging and cause collagen degradation, which causes the skin to lose its elasticity [2]. Elastin also plays a critical role in maintaining the elasticity of skin tissues after stretching and recoiling [3]. However, this elastic protein can be degraded by elastase activities. As a result, the skin is stiff if the levels of elastin in the body are reduced. Hyaluronidase breaks down hyaluronic acid, causing dry skin [4]. Tyrosinase is a copper-oxidase enzyme that initiates the production of skin pigment (melanin). Excess melanin production can lead to signs of skin aging, such as hyperpigmentation and wrinkles [5]. Due to high demand, chemicals are starting to be replaced with natural ingredients that are safer as anti-aging compounds [6]. The natural ingredient can fight oxidative stress by increasing the capacity of fibroblasts to prevent skin aging and improve skin beauty [7]. These natural ingredients can be found in plants, including native Indonesian plants.

Andaliman (*Zanthoxylum acanthopodium*) is a native plant species of the North Sumatra Province, Indonesia. The aroma of this fruit is very distinctive, resembling lemons, with a warm peppery flavor and a powerful vibrating effect on the mouth, a sensation that comes from the pericarp [8]. Andaliman grows wild in the forests of the Lake Toba Region of North Sumatra Province, Indonesia. However, this plant has begun to be cultivated by the local community [9]. As a result, there are ten varieties of andaliman fruit around the Lake Toba area: Simanuk, Sihorbo Humbang Hasundutan, Sirangkak, Variety 1, Variety 2, Sihalus, Sihorbo Samosir, Siholpu, Siganjangpat and Variety 3, each of which has different morphological characteristics [10]. In addition, each variety is characterized by certain chemical components [11]. 

Andaliman is one of the species of the *Zanthoxylum* genus. *Zanthoxylum* genus plants are mainly used as spices and medicinal agents [8] and has been reported as a potential skin anti-aging agent [12,13,14]. Activity as an anti-aging and antibacterial agent was reported after the application of 300 mg/mL of andaliman fruit ethanol extract for one month [12] and 300 mg/kg BW for two months had an impact on slowing the aging process of the brain due to D-galactose activation [13]. However, both previous anti-aging studies did not use the EOs of the andaliman fruit varieties and the molecular docking method. The bioactivity of the EOs of andaliman fruit varieties as skin anti-aging agents using the molecular docking method has not been reported. Therefore, this study aims to compare the EOs of ten andaliman fruit varieties, group them according to their varieties, and obtain the chemical components that can be used as potential skin anti-aging compounds based on molecular docking.

## 2. Materials and Methods

### 2.1. Plant Material and Sampling

**Figure 1:**
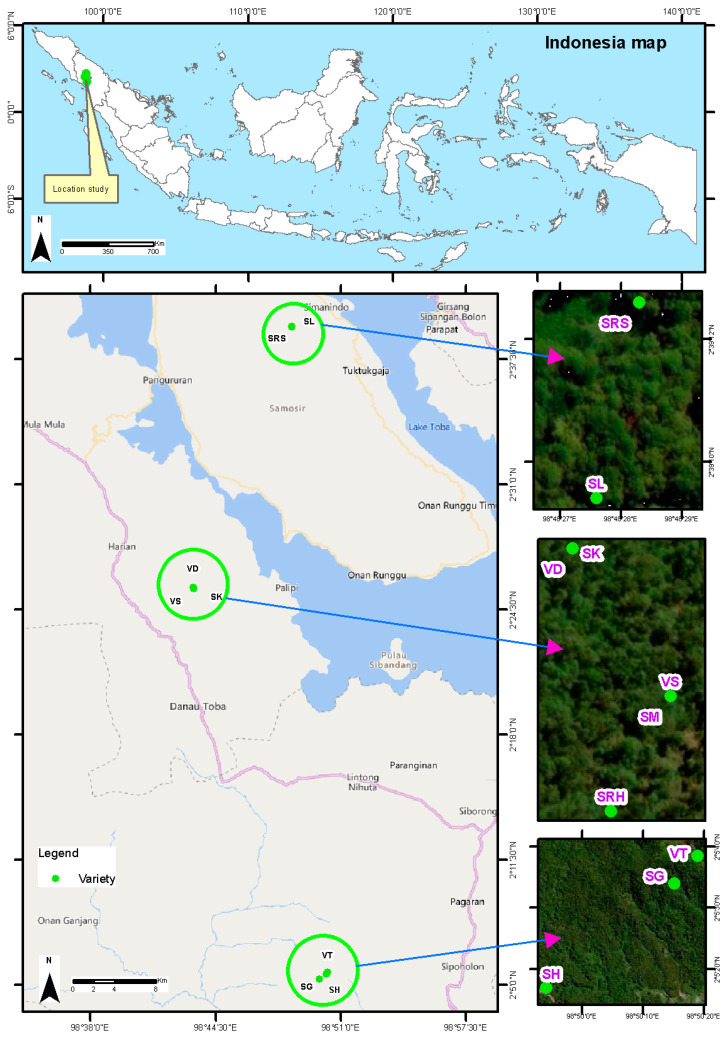
Distribution map of andaliman fruit varieties.

Andaliman fruit from ten varieties can be found in three locations in North Sumatra Province, Indonesia. Five varieties are found in the Humbang Hasundutan Regency (altitude of 1700–1800 masl): Simanuk (SM), Sihorbo Humbang Hasundutan (SRH), Sirangkak (SK), Variety 1 (VS) and Variety 2 (VD). Two varieties are found in the Samosir Regency (altitude of 1600–1700 masl): Sihalus (SL) and Sihorbo Samosir (SRS). Finally, three varieties are found in the North Tapanuli Regency (altitude of 1500–1600 masl): Siholpu (SH), Siganjangpat (SG) and Variety 3 (VT) (Figure 1).

### 2.2. Hydrodistillation

Andaliman fruit of each variety were taken from mature trees that had already produced fruit. Air-dried andaliman fruit (200 g) were collected and specified in triplicate for each variety, with a total of 30 samples. Furthermore, hydrodistillation was conducted for 6 h using the Clevenger apparatus to isolate their EOs, which were then dehydrated using sodium sulfate anhydrous pro-analysis to produce water-free andaliman fruit EOs. Before GC-MS analysis, each of andaliman EO sample was diluted with n-hexane pro-analysis, Merck. A total of 100 µL of andaliman fruit oil sample was added to 900 µL of n-hexane pro-analysis to produce samples of 1 mL. 

### 2.3. GC-MS

EOs were examined using GC-MS analysis [11]. GC-MS was conducted with Shimadzu GC-MS QP2010 Ultra (Kyoto, Japan) to apply a stationary phase of Rtx-5MS 5% diphenyl and 95% dimethyl polysiloxane (DB-5), column 30 m × 0.25 mm (Restek, Bellefonte, PA, USA). The carrier gas used was ultra-high purity helium with a pressure of 37.1 kPa, an injection volume of 1 µL, an injector temperature of 250 °C, an ion source temperature of 230 °C and an interface temperature of 230 °C in the split mode. The column was programmed from 70 °C, then increased to 230 °C with an increased rate of 10 °C/min and held for 3 min. The final temperature of the column was 270 °C with an increased rate of 5 °C/min and held for 3 min. The identification of every compound using GC-MS was conducted by matching their mass spectral profiles with the mass spectra of the data library NIST 17 (NIST, Gaithersburg, MD, USA). The retention index (RI) was determined from the retention times of C_7_–C_30_, and all *n*-alkanes were injected under similar conditions. 

### 2.4. Statistical Analysis 

A single-factor ANOVA was used to analyze the peak area value of each compound. In addition, the significant dissimilarities in the peak areas of the ten EO varieties of andaliman fruit (SM, SRH, SK, VS, VD, SL, SRS, SH, SG and VT) were elucidated using a *t*-test. Both analyses were performed using Microsoft Excel. Furthermore, we used the Orange data mining software to create a heatmap to differentiate and group the ten EOs [15].

### 2.5. Ligands and Receptor 

Based on the GC-MS analysis, the dominant and characteristic compounds of the EOs of the andaliman fruit varieties were selected as the test ligands, while the control ligand used was ascorbic acid. The ligands were downloaded from PubChem (https://pubchem.ncbi.nlm.nih.gov/) (accessed 14 January 2023) in *sdf format. The Gasteiger charge in the AutoDockTools 1.5.7 software was used to complete a portion of the 3D ligand structure, which was then saved in *pdbqt format. The Protein Data Bank (https://www.rcsb.org/) (accessed 14 January 2023) was used to obtain the three-dimensional crystal structures of hyaluronidase (2PE4), tyrosinase (5M8R), collagenase (2TCL) and elastase (3F19) in the format *pdb. Furthermore, missing residues were checked in the downloaded structure. UCSF Chimera was used to remove water and natural ligands and the prepared structures were saved in *pdb format. The AutoDockTools 1.5.7 software optimized the receptor structure and then saved it in *pdbqt format [15].

### 2.6. AdmetSAR, Molecular Docking and Visualization 

The receptor’s active site was identified to conduct targeted docking in molecular docking simulations. The suitability of the test ligand’s size and the binding area of the receptor crystal ligand is important for gridboxes. The ligands used and the config file containing the information of the gridbox were collected in a folder. AutoDock Vina was used for the molecular docking simulation. The ligand with the highest negative affinity energy was acquired from the outcome of the molecular docking analysis. The highest negative affinity energies of the ten dominant compounds and the identification of the compounds with each receptor were then analyzed and visualized by admetSAR. Ligands that were potentially significant as skin anti-aging compounds were selected from the ligands at 2 or 3 receptors. LigPlus was used for docking; then, the complex was captured on screen. AdmetSAR was used to test the selected ligands using the website http://lmmd.ecust.edu.cn/admetSAR2/ (accessed on 14 January 2023), and then testing was conducted with the advance predict option. The https://pubchem.ncbi.nlm.nih.gov (accessed on 14 January 2023) was used to obtain the SMILES ligand format used in this analysis [15].

## 3. Results

### 3.1. EO Content and Compounds of Andaliman

**Figure 2:**
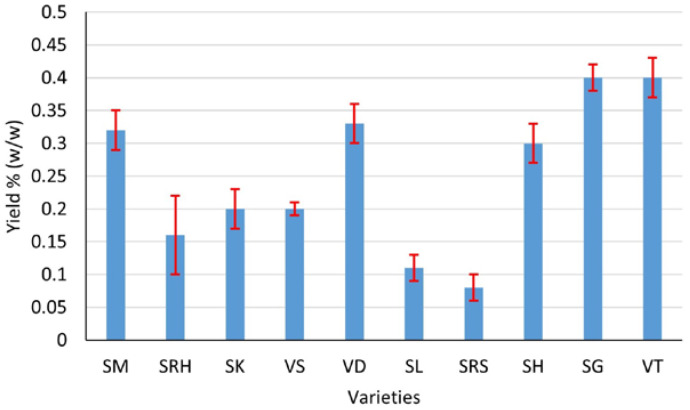
Yields of EOs of the andaliman fruit varieties SM, SRH, SK, VS, VD, SL, SRS, SH, SG and VT.

**Figure 3:**
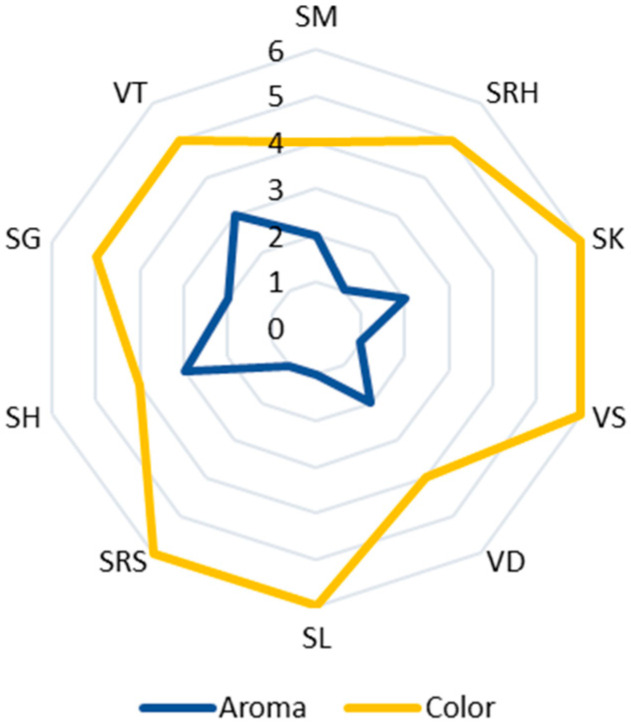
Aroma and color of the EOs of the andaliman fruit varieties. Aroma: (1) less fragrant (SRH, VS, SL and SRS); (2) fragrant (SM, SK, VD and SG); and (3) very fragrant (VT and SH). Color: (4) almost clear (SM, VD and SH); (5) light yellow (SRH, SG and VT); and (6) yellow (SK, VS, SL and SRS).

Ten varieties of andaliman fruit from the North Sumatra Province, Indonesia, have Eos with various yields, aromas and colors. Generally, the EOs of andaliman fruit yield from 0.08 to 0.4% (*w*/*w*), with the largest yield being that of Siganjangpat and Variety 3 (Figure 2). The aroma ranges from less fragrant to very fragrant, with the most fragrant aroma being that of the Siholpu and Variety 3, and the color is almost transparent, light yellow and yellow (Figure 3).

**Figure 4:**
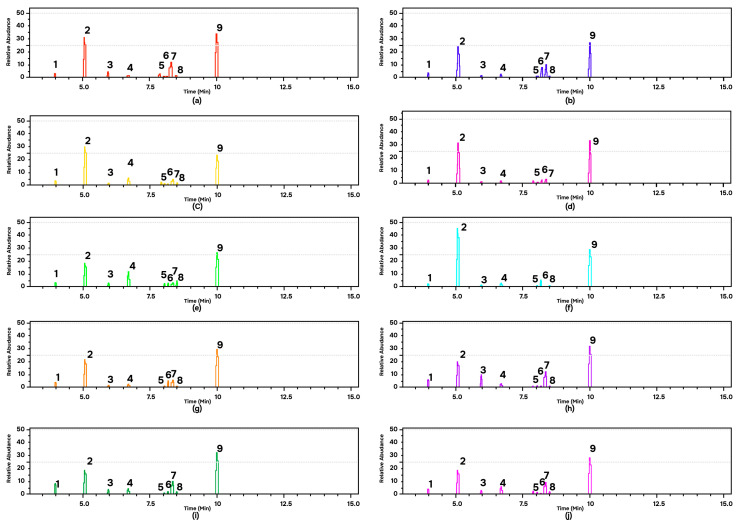
GC-MS of the EOs of andaliman fruit varieties: (**a**) SM; (**b**) SRH (**c**) SK; (**d**) VS; (**e**) VD; (**f**) SL; (**g**) SRS; (**h**) SH; (**i**) SG; and (**j**) VT. Chemical compounds: (1) alpha-Pinene; (2) D-Limonene; (3) Linalool; (4) Citronellal; (5) Neral; (6) D-Citronellol; (7) Geraniol; (8) Geranial; and (9) Geranyl acetate.

GC-MS analysis provides data on the type and abundance of chemical compounds indicated by the peak area. A higher peak area indicates a higher abundance of certain chemical components in the EOs of andaliman fruit. The chromatogram data explained that the ten varieties of andaliman fruit have almost the same main compounds, shown by the same retention time but different relative concentrations (Figure 4). Nine dominant chemical compounds in the EOs of each variety of andaliman fruit have diverse in % peak area (Table 1). All EOs dominated with D-limonene (18–45% relative area) and geranyl acetate (24–34% relative area). 

### 3.2. Heatmap

**Figure 5:**
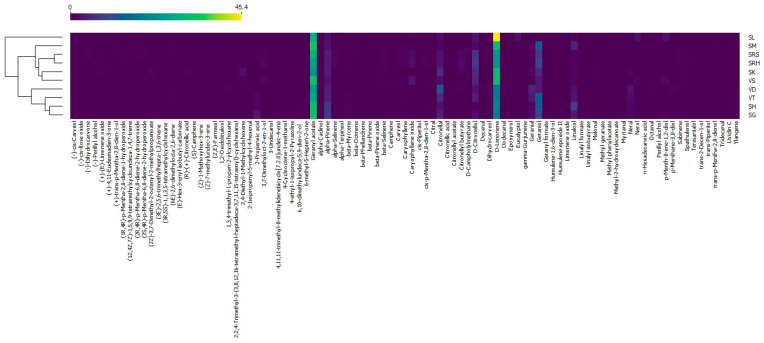
Heatmap of the andaliman fruit varieties in North Sumatra.

A heatmap was drawn using the Orange data mining software. Andaliman EOs were divided into three large clusters (Figure 5). Cluster 1 consisted of SL and was the only andaliman EO that was completely different from those of the other samples. Cluster 2 included VS, SM, SK, SRH and SRS. VS was closely related to SM and SK, while SRH was closer to SRS. SG, SH, VT and VD were grouped in Cluster 3. SG was closely related to SH, VT and VD. In addition to clustering based on the compounds, andaliman EOs were also clustered based on their origin. Cluster 3 mostly consisted of andaliman EOs that come from fruit from North Tapanuli, namely SH, SG and VT, but VD was from Humbang Hasundutan. The presence of VD was probably due to the greater abundance of D-limonene compared to the other varieties originating from Humbang Hasundutan and was more similar to those from North Tapanuli. VS, SM, SK and SRH were andaliman EOs from fruit originating from Humbang Hasundutan and were grouped in Cluster 2, with SRS originating from Samosir. The presence of SRS was also due to the abundance of D-limonene being lower than that of other varieties from Samosir and being more similar to those from Humbang Hasundutan. This result is appropriate because Humbang Hasundutan is between North Tapanuli and Samosir, so the EO content was biased. This was evidenced by VD being grouped in Cluster 3 and SRS in Cluster 2.

The color patterns on the heatmap can provide information on characteristic compounds on each andaliman EO. Cluster 1 had the highest abundance of D-limonene, characterized by a red color in the cells (Figure 5). Cluster 1, consisting of SL, was the only sample containing eucalyptol. The D-limonene abundance of cluster 2 was higher than that in cluster 3. Cluster 2 had a lower abundance of alpha-Pinene, while cluster 3 had more geraniol than the other clusters. The Sihorbo Samosir (SRS) variety was the only EO containing dihydrocarvone. The variety of Sihorbo Humbang Hasundutan (SRH) had a higher abundance of D-citronellol than the others. The highest abundance of geranyl acetate was in Simanuk (SM), while the lowest was in Sirangkak (SK). Caryophyllene oxide was most abundant in Variety 1 (VS), and geranial and nerol were abundant in Variety 2 (VD). The characteristic compounds in Variety 3 (VT) and Siholpu (SH) were geraniol and linalool, respectively. On the other hand, the variety of Siganjangpat (SG) could be distinguished because its alpha-Pinene abundance was higher than in other EOs.

### 3.3. Molecular Docking

The potential as a skin anti-aging agent is determined by the dominant chemical compound in the EOs of andaliman fruit varieties. Before molecular docking, the fourteen chemical compounds were adjusted according to the Lipinski rule and ADMET properties (Table 2). The molecular docking results are presented according to energy affinity (Table 3). Enzyme-ligand interactions were evaluated based on binding energy calculations [16].

The energy affinity value is not the only parameter supporting new drug candidate selection. The drug discovery process also considers the physicochemical properties of a compound using several parameters included in Lipinski’s rule and admetSAR. The physicochemical properties included in Lipinski’s rule are molecular weight, partition coefficient values (a log P), hydrogen donors and acceptors. The values of bioavailability, human intestinal absorption, AMES mutagenesis, carcinogenicity and enzyme inhibitors are included in the admetSAR parameters. All of the test ligands derived from the essential oils of andaliman fruit passed Lipinski’s rule. Even when tested using admetSAR parameters, all of these test ligands passed satisfactorily. These ligands have a fairly good bioavailability value, can be absorbed in the human intestinal system and do not cause mutagenesis. Linalool is the only ligand that has the potential to cause cancer. Therefore, linalool was not prioritized as a candidate skin anti-aging compound. Based on the several parameters discussed previously, geranyl acetate and D-limonene can potentially have anti-aging activity for the skin. Both compounds are abundant in the essential oil of the Sihalus variety of andaliman fruit. Considering this, the essential oil of the andaliman fruit of the Sihalus variety is a natural product that can potentially be a source of skin anti-aging compounds.

A receptor–ligand complex can be formed because of the receptors’ interaction between ligand and amino acid residues. The interaction formed can be a hydrophobic interaction and a hydrogen bond. Several of these interactions can be compared between the interactions in the receptor–ligand control complex and the receptor–ligand test complex. The value of this comparison is called the percentage similarity of the binding site (%BSS). The %BSS helps researchers to develop and predict a drug’s chemical function based on its structure, which binds to the receptor [17].

**Figure 6:**
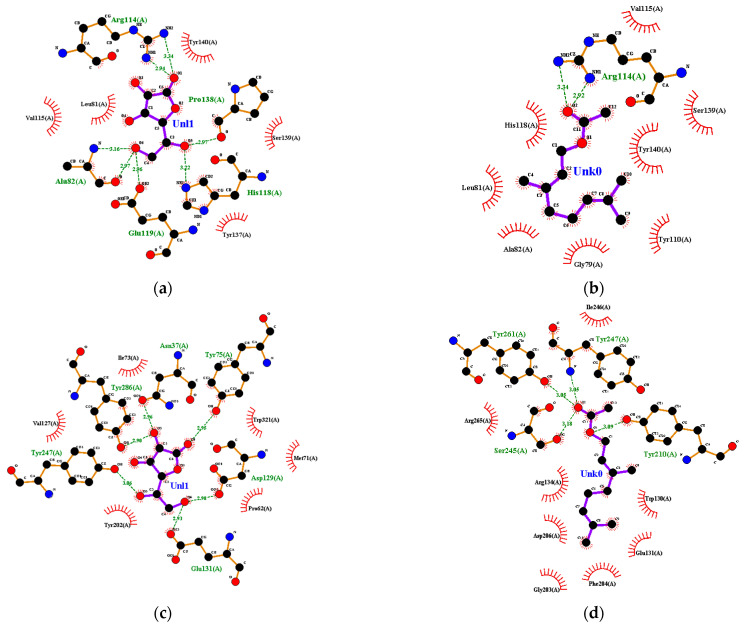

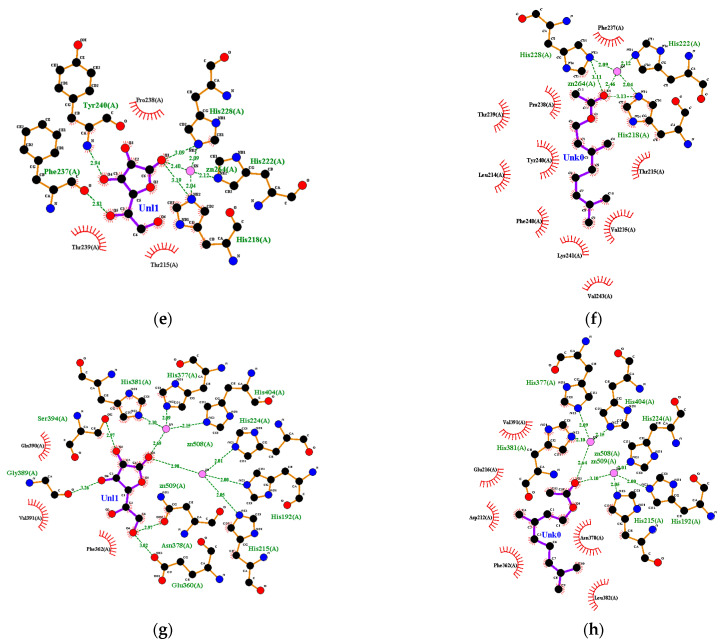
Ligand–receptor complex interaction visualization of: (**a**) ascorbic acid–collagenase, (**b**) geranyl acetate–collagenase, (**c**) ascorbic acid–hyaluronidase, (**d**) geranyl acetate–hyaluronidase, (**e**) ascorbic acid–elastase, (**f**) geranyl acetate–elastase, (**g**) ascorbic acid–tyrosinase and (**h**) geranyl acetate–tyrosinase.

Ascorbic acid had a hydrophobic interaction with amino acid residues on collagenase, including Leu-81 and Ser-139. On the other hand, ascorbic acid had hydrogen bonds with Ala-82 and Pro-138 (Figure 6a). Geranyl acetate had a %BSS of 70% because it interacted with seven amino acid residues, including Leu-81 and Ser-139, which interacted with ascorbic acid (Figure 6b). Oxygen atoms in geranyl acetate hydrogen-bonded with two nitrogen atoms in Arg-114 in collagenase, and the distance between them was 2.92 and 3.34 Å (Figure 6b), respectively.

Ile-73, Val-127, Tyr-202 and Trp-321 from hyaluronidase interacted hydrophobically with ascorbic acid. Other amino acid residues, including Asp-129, Glu-131, Tyr-247 and Tyr-286 from hyaluronidase, had a hydrogen bond with ascorbic acid (Figure 6c). Several amino acids, such as Asp-129, Glu-131, Tyr-202 and Trp-321, play a critical role in hyaluronidase’s active site. Geranyl acetate had hydrophobic interactions with the amino acid residues Trp-130, Glu-131, Arg-134, Gly-203, Phe-204, Asp-206, Ile-246 and Arg-265, and hydrogen-bonded with Tyr-210, Ser-245, Tyr-247 and Tyr-261 at a distance of 3.09, 3.18, 3.05 and 3.05 Å, respectively (Figure 6d). The %BSS for geranyl acetate ligand was 17%.

Ascorbic acid had a hydrophobic interaction with amino acid residues from elastase, including His-228, Phe-237 and Tyr-240. Ascorbic acid was also hydrogen-bonded with several amino acid residues from elastase, including Thr-215 and Pro-238 (Figure 6e). Geranyl acetate interacted hydrophobically with Leu-214, Thr-215, Val-235, Phe-237, Pro-238, Thr-239, Tyr-240, Lys-241 and Val 243, and it also had a complex hydrogen bond with Zn, His-218, His-222 and His-228 of elastase (Figure 6f). Geranyl acetate had 100%BSS because it had eight interactions that occurred in the ascorbic acid–elastase interaction. Hydrogen bonds formed between ascorbic acid and some amino acid residues from tyrosinase, such as His-215, Glu-360, Asn-378 and Gly-389, as well as Phe-362, Glu-390 and Val-391, interacted hydrophobically in tyrosinase (Figure 6g). The %BSS for geranyl acetate was 50% (Figure 6h).

## 4. Discussion

The yield of andaliman fruit EOs from North Sumatra, Indonesia, is equivalent to that of research on the yield of EOs of andaliman fruit reported from Yunnan Province, China, yielding 0.36%, with 63 chemical components and the main components identified as estragole (15.46%), followed by eucalyptol (10.94%) and β-caryophyllene (5.52%) [18]. The most dominant chemical compound of andaliman fruit from Indonesia in this study is geranyl acetate, a monoterpene. Geranyl acetate, which has a pleasant floral or fruity rose aroma, was also reported as the main component in previous studies; only the concentrations differed [19]. Differences in altitudinal origin can influence differences in chemical content [20].

Testing the physicochemical properties of a compound that has potential as a skin anti-aging agent is carried out before the molecular docking process. The physicochemical properties of compounds were tested using Lipinski’s rules of five and admetSAR parameters. Lipinski’s rules measure drug likeness in small molecules [21] and demonstrate a ligand’s solubility and permeability in the drug development process [22]. The parameters included in Lipinski’s rules are molecular weight of <500 g/mol, partition coefficient (a log P) of <5, number of hydrogen donors, and acceptors of <5 and <10. Drug candidate compounds must have a molecular weight of <500 g/mol to avoid negative effects caused by build-up because large molecules tend not to pass through the membrane by passive diffusion [23]. The solubility of a compound predicts the hydrophobicity strength of a compound and is described in coefficient partition (a log P). Compounds with high partition coefficient values will be left in the lipid bilayer membrane to increase the hydrophobicity strength [24]. The hydrogen bonds formed are related to the number of hydrogen donors and acceptors. This can cause a compound to interact strongly with a polar environment, inhibiting the absorption process from reaching the receptor [25]. All ligands that have skin anti-aging potential passed Lipinski’s rule test.

The admetSAR test was conducted in this study to complete the physicochemical property testing of compounds that have potential as skin anti-aging agents. AdmetSAR is an acronym for absorption, distribution, metabolism, excretion, toxicity and structure–activity relationship; therefore, it describes the compound’s behavior starting from the moment it enters the human body and the influence of the structure of these compounds on their activities. Pharmacokinetic and pharmacodynamic analyses are included in this test because they help researchers, especially in the drug industry, to predict and understand the behavior of drug candidates, such as their half-life, mechanism, efficacy and safety in the human body. The admetSAR parameters considered in this study were human intestinal absorption (HIA), AMES mutagenesis, carcinogenicity and enzyme inhibitors. Enzyme inhibitors show the strength of the compound against the enzyme.

Most of the tested ligands’ affinity energies in the hyaluronidase, elastase and tyrosinase complexes had more negatives than the control ligands. This indicated that the tested ligand could inhibit the skin’s anti-aging receptor. These findings are aligned with previous skin anti-aging studies using ginger rhizome as a natural product [15]. However, regarding the collagenase complex in this study, all of the tested ligands had a more positive energy affinity than ascorbic acid, so this enzyme seems unsuitable for use as a pathway to inhibit the skin-aging process. In contrast, in other studies, the highest negative energy affinity appeared in four receptor complexes, including collagenase [15]. The (-)-dihydrocarvone had the most negative average energy affinity, which was −6.35 kcal/mol (Table 2). The next most negative average energy affinity was −6.25 kcal/mol by geranyl acetate and D-limonene (Table 2). Finally, caryophyllene oxide had an average energy affinity of −6.23 kcal/mol and tended to inhibit 5M8R specifically because its energy affinity was the most negative compared to other test ligands in the same complex, −7.20 kcal/mol (Table 2).

The selection of compounds that have potential as skin anti-aging agents also considers the abundance of the compounds in andaliman EOs. Unfortunately, the abundances of (-)-dihydrocarvone and caryophyllene oxide were low and not found in some andaliman EOs, so they lack potential as skin anti-aging compounds. Geranyl acetate and D-limonene had relatively high abundances in all andaliman EOs, and their energies affinity were negative enough to inhibit the aging processes. Based on the molecular docking analysis, geranyl acetate and D-limonene have the highest potential as skin anti-aging compounds in andaliman EOs. Geranyl acetate was abundant in SM, whereas D-limonene was abundant in SL. If the abundances of these two compounds were accumulated, then the best andaliman EO, which had the highest abundance of skin anti-aging compounds, was Sihalus (SL). Therefore, the Sihalus andaliman EO, derived from Samosir, has the greatest potential as skin anti-aging agent. 

Based on their components, especially limonene, other species of the genus *Zanthoxylum*, including Japanese pepper (*Zanthoxylum piperitum*) [26], Chinese prickly ash (*Zanthoxylum bungeanum*) [27] and *Zanthoxylum schinifolium* [28], are predicted to have the same anti-aging potency. *Z. piperitum* essential oil is dominated with a limonene content with the potency to inhibit hyaluronidase, elastase and tyrosinase [29]. *Z. bungeanum* and *Z. schinifolium* are also high in limonene [30,31].

## 5. Conclusions

There are 97 chemical components in the EOs of the ten varieties of andaliman fruit, with the main chemical components being geranyl acetate, D-limonene, geraniol, α-Pinene, citronellal, D-citronellol, linalool, geranial and neral. The ten varieties of andaliman fruit EOs were grouped into three clusters. The geranyl acetate and D-limonene compounds found in the EOs of andaliman fruit have the potential to be anti-aging skin compounds. Both compounds were abundant in the Sihalus variety of andaliman fruit. Therefore, the EO of the Sihalus variety of andaliman fruit has the most potential as a skin anti-aging agent. Therefore, it can be developed for applications in the pharmaceutical industry. However, this research was limited to in silico studies; it is essential to perform in vitro, in vivo and clinical studies to prove this. 

## Figures and Tables

**Table 1:** The main compounds of EOs of andaliman fruit varieties.

Compounds	Retention Index (RI)	% Peak Area									
		SM	SRH	SK	VS	VD	SL	SRS	SH	SG	VT
α-pinene	948.4	3.00 ± 0.03	4.02 ± 0.82	4.60 ± 0.04	3.24 ± 0.44	4.45 ± 0.01	2.71 ± 0.02	4.51 ± 0.66	7.76 ± 0.10	8.28 ± 0.11	4.54 ± 0.03
D-limonene	1033.3	31.61 ± 0.12	24.29 ± 2.68	30.71 ± 0.55	31.66 ± 8.22	18.27 ± 0.21	45.40 ± 0.28	23.87 ± 1.12	20.71 ± 0.48	19.24 ± 0.39	19.18 ± 0.40
linalool	1086.4	5.22 ± 0.04	1.15 ± 0.06	1.64 ± 0.02	1.20 ± 0.27	2.49 ± 0.01	1.01 ± 0.02	1.10 ± 0.11	9.33 ± 0.08	3.99 ± 0.06	3.21 ± 0.04
citronellal	1230.3	1.14 ± 0.02	3.11 ± 0.58	6.29 ± 0.06	2.63 ± 0.62	12.23 ± 0.03	2.32 ± 0.02	2.77 ± 0,65	3.04 ± 0.07	4.96 ± 0.02	6.22 ± 0.04
neral	1240.7	0.52 ± 0.01	0.18 ± 0.16	1.00 ± 0.02	0.12 ± 0.10	3.17 ± 0.01	0.20 ± 0.09	0.09 ± 0.16	1.28 ± 0.02	1.45 ± 0.02	2.20 ± 0.02
D-citronellol	1251.4	2.02 ± 0.01	8.23 ± 2.44	6.39 ± 0.24	5.68 ± 1.79	3.31 ± 0.02	3.16 ± 0.02	6.88 ± 2.49	1.00 ± 0.04	2.66 ± 0.06	4.74 ± 0.39
geraniol	1260.1	12.92 ± 0.07	10.09 ± 3.74	5.52 ± 0.03	3.53 ± 3.26	3.77 ± 0.02	nd	7.98 ± 3.83	13.31 ± 0.01	10.48 ± 0.10	14.53 ± 0.06
geranial	1276.3	1.24 ± 0.02	0.52 ± 0.45	1.85 ± 0.04	nd	5.15 ± 0.02	0.28 ± 0,24	0.52 ± 0.45	1.50 ± 1.30	2.44 ± 0.02	3.69 ± 0.03
geranyl acetate	1371.5	34.51 ± 0.26	27.25 ± 5.18	24.51 ± 0.22	33.16 ± 1.91	26.01 ± 0.04	29.01 ± 0.18	30.41 ± 5.29	32.41 ± 0.34	32.94 ± 0.23	28.43 ± 0.14

nd: not detected.

**Table 2:** Physicochemical parameters and ADMET test ligands.

Ligand	Physicochemical Parameters					ADMET Parameters				
	Molecular Weight (<500 g mol^−1^)	a Log P (<5)	Hidrogen Bonds Donor (<5)	Hidrogen Bonds Acceptor (<10)	Information	Bioavailability (Score)	Human Intestinal Absorption (HIA)	AMES Mutagenesis	Carcinogenicity	Enzyme Inhibitor
α-Pinene	136.24	3.00	0	0	Pass	GB (0.55)	HIA (+)	AMES (−)	NC	−0.34
D-Limonene	136.24	3.31	0	0	Pass	GB (0.55)	HIA (+)	AMES (−)	NC	−0.21
Linalool	196.29	3.24	0	2	Pass	GB (0.55)	HIA (+)	AMES (−)	C	0.36
Citronellal	154.25	2.96	0	1	Pass	GB (0.55)	HIA (+)	AMES (−)	NC	−0.03
Neral	152.24	2.88	0	1	Pass	GB (0.55)	HIA (+)	AMES (−)	NC	0.02
D-Citronellol	156.27	2.75	1	1	Pass	GB (0.55)	HIA (+)	AMES (−)	NC	−0.12
Geraniol	154.25	2.67	1	1	Pass	GB (0.55)	HIA (+)	AMES (−)	NC	0.28
Geranial	152.24	2.88	0	1	Pass	GB (0.55)	HIA (+)	AMES (−)	NC	0.02
Nerol	154.25	2.67	1	1	Pass	GB (0.55)	HIA (+)	AMES (−)	NC	0.28
Eucalyptol	154.25	2.74	0	1	Pass	GB (0.55)	HIA (+)	AMES (−)	NC	−0.15
(-)-Dihydrocarvone	152.24	2.57	0	1	Pass	GB (0.55)	HIA (+)	AMES (−)	NC	−0.31
Geranyl acetate	196.29	3.24	0	2	Pass	GB (0.55)	HIA (+)	AMES (−)	NC	0.21
Caryophyllene oxide	220.36	3.94	0	1	Pass	GB (0.55)	HIA (+)	AMES (−)	NC	0.57

**Table 3:** Molecular docking of the EOs of the andaliman fruit varieties.

Ligand	Energy Affinity (kcal/mol) in Target Protein			
	2TCL	2PE4	3F19	5M8R
α-Pinene	−5.7	−5.3	−5.7	−5.5
D-Limonene	−5.9	−6.5	−6.6	−6.0
Linalool	−5.6	−5.9	−6.1	−4.8
Citronellal	−5.0	−5.2	−6.1	−5.3
Neral	−5.1	−5.8	−5.9	−5.2
D-Citronellol	−4.8	−5.6	−6.1	−4.8
Geraniol	−5.8	−5.6	−6.3	−4.9
Geranial	−5.3	−6.1	−6.4	−6.0
Nerol	−5.4	−5.6	−6.4	−5.1
Eucalyptol	−5.6	−5.3	−6.1	−5.5
(-)-Dihydrocarvone	−6.1	−6.7	−6.9	−5.7
Geranyl acetate	−6.0	−5.9	−7.1	−6.0
Caryophyllene oxide	−5.3	−6.1	−6.3	−7.2
Ascorbic acid	−6.4	−5.6	−6.1	−6.0

## Data Availability

Data are contained within the article.
